# Development and Validation of a Highly Sensitive LC–MS/MS Method for the Precise Quantification of Sitagliptin in Human Plasma and Its Application to Pharmacokinetic Study

**DOI:** 10.3390/molecules30142995

**Published:** 2025-07-16

**Authors:** Yuna Song, Wang-Seob Shim, Eunseo Song, Yebeen Park, Bo-Hyung Kim, Sangmin Lee, Eun Kyoung Chung, Kyung-Tae Lee

**Affiliations:** 1 Department of Biomedical and Pharmaceutical Science, Graduate School, Kyung Hee University, 26, Kyungheedaero, Dongdaemun-gu, Seoul 02447, Republic of Korea; songyu0819@khu.ac.kr (Y.S.); sssk2303@khu.ac.kr (E.S.); yeebin1028@khu.ac.kr (Y.P.); leesm@khu.ac.kr (S.L.); cekchung@khu.ac.kr (E.K.C.); 2 Kyung Hee Drug Analysis Center, College of Pharmacy, Kyung Hee University, 26, Kyungheedaero, Dongdaemun-gu, Seoul 02447, Republic of Korea; wsshimm@khu.ac.kr; 3 Department of Clinical Pharmacology and Therapeutics, Kyung Hee University Hospital, 23, Kyungheedaero, Dongdaemun-gu, Seoul 02447, Republic of Korea; bhkim98@khu.ac.kr; 4 Department of Pharmacy, College of Pharmacy, Kyung Hee University, 26, Kyungheedaero, Dongdaemun-gu, Seoul 02447, Republic of Korea

**Keywords:** sitagliptin, human plasma, bioanalytical method validation, pharmacokinetics, dipeptidyl peptidase-4 inhibitor, LC-MS/MS

## Abstract

Sitagliptin is an orally bioavailable selective DPP4 inhibitor that reduces blood glucose levels without significant increases in hypoglycemia. The aim of this study was to design and validate an innovative, rapid, and highly sensitive LC–MS/MS assay for the precise measurement of sitagliptin concentrations in human plasma. This analytical method, utilizing sitagliptin-d4 as the internal standard, is performed using only 100 μL of plasma and a liquid–liquid extraction procedure based on methyl tert-butyl ether (MTBE). Chromatographic separation is expertly achieved with a Kinetex^®^ C18 column under isocratic elution, employing a perfect 1:1 blend of 5 mM ammonium acetate (with 0.04% formic acid) and acetonitrile, and maintaining an efficient flow rate of 0.2 mL/min. Detection occurs in positive ionization mode through multiple reaction monitoring, precisely targeting transitions of *m*/*z* 408.2 → 193.0 for sitagliptin and 412.2 → 239.1 for the IS. The total runtime of this assay is under 2 min. Comprehensive validation in line with MFDS and FDA criteria demonstrates outstanding linearity (5–1000 ng/mL, r^2^ > 0.998), alongside impressive levels of accuracy, precision, recovery and sample stability. Due to its minimal sample requirement and high-throughput capability, the validated approach is highly appropriate for pharmacokinetic and bioequivalence assessments involving sitagliptin.

## 1. Introduction

Type 2 diabetes mellitus (T2DM) is a complex metabolic disorder marked by consistently high blood sugar levels, primarily caused by insulin resistance and impaired function of pancreatic β-cells [1]. As the global prevalence of T2DM continues to increase, there is an urgent need for effective treatments that can manage blood glucose levels while minimizing side effects. Among the various classes of oral antidiabetic medications, dipeptidyl peptidase-4 (DPP-4) inhibitors have gained recognition for their ability to enhance insulin secretion and suppress glucagon release in a manner dependent on glucose levels, which helps reduce the risk of hypoglycemia [2].

Sitagliptin, a well-known DPP-4 inhibitor, works by blocking the breakdown of incretin hormones, particularly glucagon-like peptide-1 (GLP-1) [3]. This mechanism enhances insulin secretion in response to glucose. It belongs to the chemical class of 1,2,4-triazolo[4,3-a]pyrazines and is structurally identified as 7-[(3R)-3-amino-1-oxo-4-(2,4,5-trifluorophenyl)butyl]-5,6,7,8-tetrahydro-3-(trifluoromethyl) [4]. Sitagliptin has been approved for use as both a monotherapy and in combination therapy for patients with T2DM [5]. Additionally, it demonstrates a high preference for DPP-4 over similar enzymes such as DPP-8 and DPP-9, which contributes to its favorable safety and tolerability profile [6].

Pharmacokinetic investigations reveal that sitagliptin possesses a high oral bioavailability of approximately 87% and attains its peak plasma concentration (*C*_max_) within 1 to 4 h post-administration. Its terminal half-life is estimated to be between 10 and 12 h, and it demonstrates moderate binding (38%) to plasma proteins [7,8]. The drug’s apparent volume of distribution, approximately 198 L, indicates extensive distribution within tissues [7,9]. Sitagliptin is predominantly eliminated through renal excretion in its unchanged form, while hepatic metabolism—primarily via CYP3A4 and CYP2C8—accounts for a minor fraction (<16%) of total clearance [10,11]. Consequently, there is a low likelihood of CYP-mediated drug-drug interactions [6]. In patients with renal impairment, systemic exposure to sitagliptin increases in accordance with the severity of the dysfunction, necessitating appropriate dose adjustments [12].

Various analytical platforms have been investigated for the quantification of sitagliptin in diverse biological matrices, with high-performance liquid chromatography (HPLC), ultra-performance liquid chromatography (UPLC), and liquid chromatography-tandem mass spectrometry (LC-MS/MS) emerging as prevalent methodologies due to their analytical precision. Earlier HPLC-based methods required substantial sample volumes (>800 μL) and exhibited extended run times (>10 min), which limited throughput [13]. Although LC-MS/MS provides enhanced sensitivity, many existing protocols still necessitate large plasma volumes (up to 1000 μL) to achieve lower limits of quantification [3,14]. Thus, our objective was to develop a reliable, faster, and high throughput method with simple LLE extraction preparation for the routine determination of sitagliptin in human plasma. The analytical method described in this study was a robust LC-MS/MS technique that requires only 100 μL of plasma, achieving a lower limit of quantification (LLOQ) of 5 ng/mL and a total run time of less than 2 min. Consequently, this study aimed to create a sensitive and rapid analytical method capable of assessing the marketed pharmaceutical formulations of 100 mg sitagliptin tablet in Koreans.

## 2. Results and Discussion

### 2.1. Method Development

#### 2.1.1. Mass Spectrometry

**Figure 1:**
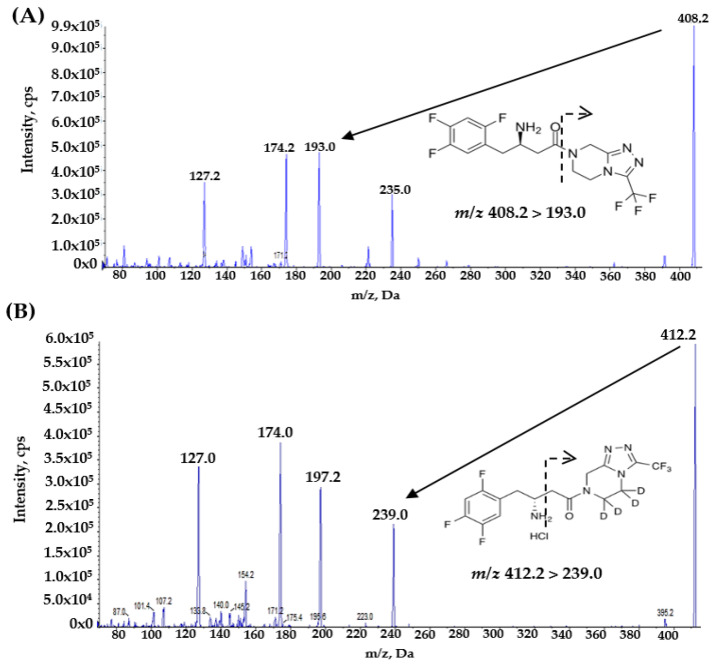
Ion product mass spectra and fragmentation structural patterns of (**A**) sitagliptin and (**B**) sitagliptin-d4.

To enhance detection sensitivity in mass spectrometry analysis, sitagliptin and the internal standard were introduced into the system via direct methanol infusion at a controlled rate of 10 μL/min delivered by a syringe pump. Following the assessment of ionization options, positive-mode electrospray ionization was implemented due to its demonstrated effectiveness in generating robust signals for sitagliptin molecules, aligning with findings from earlier published research [15,16]. Under these optimized conditions, consistent formation of product ions was achieved. Quantitative analysis utilized multiple reaction monitoring (MRM) targeting the primary mass transition of *m*/*z* 408.2 → 193.0 for sitagliptin and *m*/*z* 412.2 → 239.0 for IS (Figure 1). These specific fragmentation pathways were validated through preliminary Q1 full-scan experiments before being employed for quantification purposes.

#### 2.1.2. Chromatographic Conditions

Multiple reversed-phase chromatographic columns underwent evaluation to determine the optimal separation characteristics for sitagliptin and its internal standard. The assessment included various stationary phases such as Halo^®^ Phenyl-Hexyl (Advanced Materials Technology, Wilmington, DE, USA), Gemini 5u C18 (Phenomenex, Torrance, CA, USA), and additional C18-based materials. Following comprehensive testing, the Kinetex C18 column manufactured by Phenomenex (dimensions: 100 × 2.1 mm, particle size: 2.6 μm) was selected as the optimal choice due to its fine chromatographic performance metrics, including enhanced peak symmetry, detection sensitivity, and consistent results across sequential analyses. The chromatographic system employed an isocratic mobile phase comprising equal proportions of two solutions: aqueous 5 mM ammonium formate containing 0.04% formic acid (solvent A) and pure acetonitrile (solvent B). During method development, an unexpected signal reduction was identified at elevated concentrations, which was subsequently resolved through precise adjustment of the formic acid concentration in the aqueous component. The optimized conditions established through this development process delivered stable retention behavior and improved peak geometry for both the target compound and the internal standard, facilitating reliable quantitative analysis.

#### 2.1.3. Plasma Sample Preparation

Numerous techniques for preparing samples have been explored to extract sitagliptin from plasma, including liquid–liquid extraction (LLE) [16,17,18], solid-phase extraction (SPE) [19], and protein precipitation (PP) [3,14,20,21,22,23,24,25]. The analysis method for each reference is summarized and attached as Supplementary Data (Table S1). After careful consideration, SPE was ultimately ruled out due to its higher costs and lengthy processing times, while PP demanded larger plasma volumes. As a result, LLE became the preferred method for its operational simplicity and reasonable recovery rates. Among the solvents tested, MTBE alone demonstrated remarkable efficacy, achieving a recovery rate of approximately 79.9–85.3%. During method optimization, the initial reconstitution solvent—a blend of 50% acetonitrile and 0.04% formic acid in 5 mM ammonium formate (1:1, *v*/*v*)—proved to diminish peak intensity at elevated analyte concentrations. In refining this approach, the reconstitution solvent was artfully adjusted to a 1:1 (*v*/*v*) mixture of 0.1% formic acid in distilled water and 100% acetonitrile, resulting in more consistent and robust peak areas. The reconstitution volume was set to 1 mL, followed by the injection of 5 μL from each reconstituted sample. It is noteworthy that previous studies typically employed reconstitution volumes ranging from 500 to 1000 μL, with an injection volume of 10 μL [3,16,25]. In contrast, this study embraced a higher reconstitution volume paired with a reduced injection amount, a strategic choice that not only enhanced sensitivity but also conserved samples.

### 2.2. Method Validation

#### 2.2.1. Selectivity and Lower Limit of Quantitation (LLOQ)

**Figure 2:**
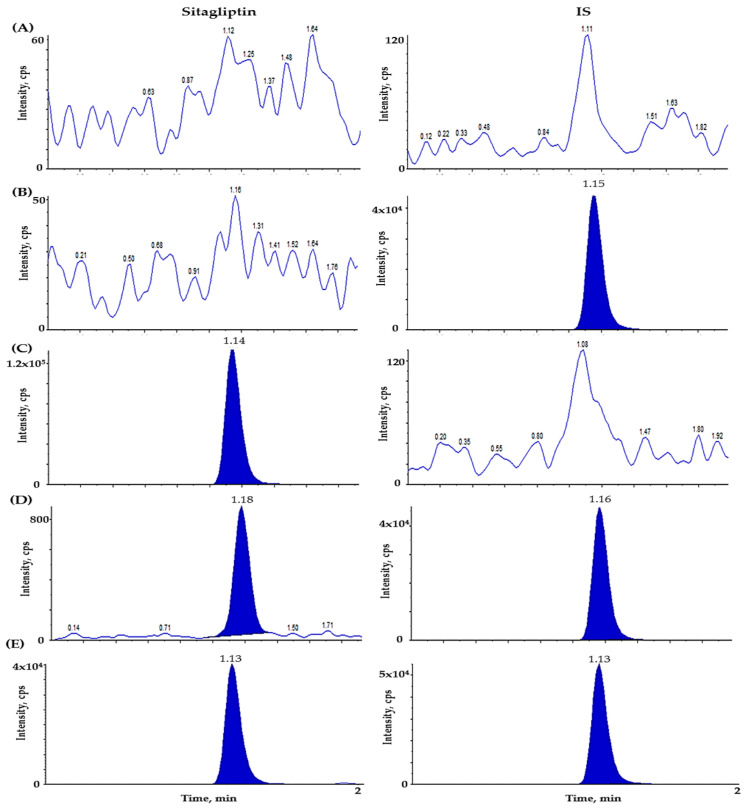
Illustrates chromatograms of the following plasma samples (**A**) double blank plasma (devoid of both sitagliptin and internal standard), (**B**) blank plasma spiked with IS (1000 ng/mL), (**C**) blank plasma spiked with sitagliptin (ULOQ, 1000 ng/mL), (**D**) blank plasma spiked with sitagliptin (LLOQ, 5 ng/mL) and IS (1000 ng/mL), (**E**) samples in human plasma at 2 h after oral administration of sitagliptin.

To assess selectivity, plasma samples from six different human donors were pooled. As illustrated in Figure 2, we obtained representative chromatograms from a variety of conditions: blank human plasma, plasma infused with sitagliptin-d4 (IS, 1000 ng/mL), plasma spiked with sitagliptin at 1000 ng/mL, plasma featuring both sitagliptin and IS (5 ng/mL and 1000 ng/mL), and a sample collected 2 h after the oral administration of sitagliptin (100 mg) in humans, later spiked with the internal standard. Our method displayed no significant endogenous interference at the retention times of both sitagliptin and the internal standard, highlighting the robustness of the approach. Additionally, we validated a lower LLOQ of 5 ng/mL, achieving an impressive signal-to-noise ratio (S/N) exceeding 10, which speaks to the method’s sensitivity. This outcome was reproduced with previously reported detection thresholds in the literature. Notably, the LLOQ established in this study reflects the values found in earlier research, affirming that we have successfully attained a level of analytical sensitivity that meets the highest standard [16].

#### 2.2.2. Linearity and Carryover

The calibration curve of sitagliptin in this study demonstrated acceptable linearity across a wide concentration range of 5–1000 ng/mL. All calibration levels yielded a correlation coefficient (*r*^2^) exceeding 0.998, while the coefficient of variation (CV) for the regression slope remained impressively low at 1.97%, underscoring the method’s outstanding reproducibility (Table 1). In a prior study [14], linearity was observed over a more limited range of 10–500 ng/mL, with *r*^2^ surpassing 0.999. In stark contrast, the current method not only achieved superb linearity and minimal variability but also encompassed a broader range that includes LLOQ. This enhancement indicates a significant improvement in analytical efficiency while preserving both sensitivity and accuracy. Collectively, these findings strongly support the assertion that this validated method is both reliable and well-suited for pharmacokinetic profiling and bioequivalence testing. Furthermore, it is noteworthy that no carryover was detected for either sitagliptin or IS at the upper limit of quantitation (ULOQ) and LLOQ concentrations in human plasma.

#### 2.2.3. Precision and Accuracy

Evaluations of precision and accuracy for sitagliptin were performed using five replicate measurements at four concentration levels: LLOQ (5 ng/mL), LQC (15 ng/mL), MQC (300 ng/mL), and HQC (800 ng/mL), assessed under both intra- and inter-day settings. These measurements were assessed under both intra-day and inter-day conditions. The intra-day coefficients of variation ranged from 1.52% to 3.72%, while inter-day variation ranged from 1.81% to 9.87%. Accuracy values for intra-day analyses were between 95.70% and 105.94%, and for inter-day analyses, they ranged from 97.20% to 100.23% (Table 2). All outcomes satisfied the acceptance criteria for precision and accuracy, as established by the MFDS and the USFDA bioanalytical method validation guidelines [26,27].

#### 2.2.4. Recovery and Matrix Effect

Table 3 shows the extraction recovery and matrix effect of sitagliptin and IS at a concentration of 1000 ng/mL across three QC concentrations (15, 300, and 800 ng/mL, *n* = 6 from 6 different human donors). The recovery rates at each QC level, as well as for the IS, were determined by comparing the mean peak areas of the extracted samples with those of equivalent aqueous standard solutions. The average recovery of sitagliptin ranged from 79.51% to 83.20%, with %CV values ranging from 1.69% to 5.22%. The IS showed an average recovery of 83.42%, with a %CV of 2.91%. According to the prior report [16], when MTBE and dichloromethane (80:20, *v*/*v*) were used as extraction solvents, the mean recovery for sitagliptin at the LQC, MQC, and HQC levels was 68.1 ± 1.1%. In the same study, fluoxetine was used as the internal standard, with a recovery of only 54.1 ± 1.0%, which was significantly lower than the deuterated analog (sitagliptin-d4) employed in the present study. These results demonstrate that the current method achieved superior recovery for both the analyte and IS, with minimal variation between them, supporting its efficiency and reliability.

To assess the matrix effect, the peak areas of sitagliptin and IS introduced into extracted blank human plasma were compared with those from equivalent aqueous standard solutions. The matrix effect for sitagliptin ranged from 104.64% to 107.30%, with %CV values between 1.38% and 6.42%, while that for the IS was 103.04%, with a %CV of 2.59%. In a previous study [17], MTBE was also used as the extraction solvent, and matrix effects were evaluated at low (5 ng/mL) and high (200 ng/mL) concentrations. The reported matrix effects were 102.5% at low and 80.09% at high concentrations, suggesting potential ion suppression at higher concentrations due to endogenous interference. In contrast, the present study demonstrated matrix effects close to 100% across all QC levels, with low %CV values, indicating minimal matrix interference and consistent ionization efficiency. The recovery values of sitagliptin and IS in this study (79.51–83.20% and 83.42%, respectively) were superior to those reported in previous studies. Moreover, the observed matrix effects were consistently close to 100% and within acceptable limits, supporting the robustness of the developed method for precise quantification in biological matrices. The ICH M10 guidelines, released on 24 May 2022, specify that the matrix effect should be evaluated three times at concentrations of 15 and 800 ng/mL from six distinct sources. However, the authors conducted the study only once at concentrations of 15, 300, and 800 ng/mL, rather than in triplicate.

#### 2.2.5. Stability

Table 4 reveals a compelling assessment of sitagliptin’s stability under various conditions, highlighting its robustness across stock solutions, working solutions, and human plasma. Stock solutions of sitagliptin stored at room temperature (~25 °C) for just 3 h maintained peak areas for both low and high QC levels within an impressive range of 99.25% to 100.89% when compared to freshly prepared samples. Furthermore, working solutions kept at room temperature for 7 h displayed mean peak area values between 95.93% and 100.19% for both sitagliptin and the IS, reflecting minimal degradation during this brief storage period. The investigation of sitagliptin’s stability in human plasma under a variety of storage conditions yielded equally encouraging results. Plasma samples stored at room temperature for 7 h retained between 96.99% and 102.57% of their initial concentration. Similar stability was observed after 7 h at 4 °C (ranging from 96.29% to 102.57%) and at –70 °C (with values between 96.77% and 99.61%). Moreover, following three freeze–thaw cycles, concentrations remained steadfast, ranging from 97.71% to 103.73%. For samples processed through LLE and kept in an autosampler at 10 °C for 30 h, the stability was noteworthy, showing values from 96.83% to 105.20%. Given that all variations fell within the acceptable ±15% threshold, it is evident that sitagliptin stands as a stable compound across all tested conditions, whether in plasma, stock solutions, or working solutions. This stability underscores its potential for reliable application in various therapeutic contexts. Nonetheless, further studies are needed to analyze sitagliptin in lipid-soluble and diabetic plasma.

### 2.3. Application to a Bioequivalence Study

In this study, we embarked on an exploration of the pharmacokinetic characteristics of sitagliptin in a group of eight healthy Korean male volunteers, following the oral administration of a 100 mg sitagliptin tablet. Previous clinical studies have elegantly delineated the pharmacokinetic parameters for sitagliptin at this dosage, reporting a maximum concentration (*C*_max_) of 327.98 ng/mL, a time to reach maximum concentration (*T*_max_) of 2.68 h, and a terminal half-life (*T*_1/2_) of 8.92 h [20]. Our findings harmoniously align with these earlier results, underscoring the robustness of our study design and the precision of the analytical methods employed.

**Figure 3:**
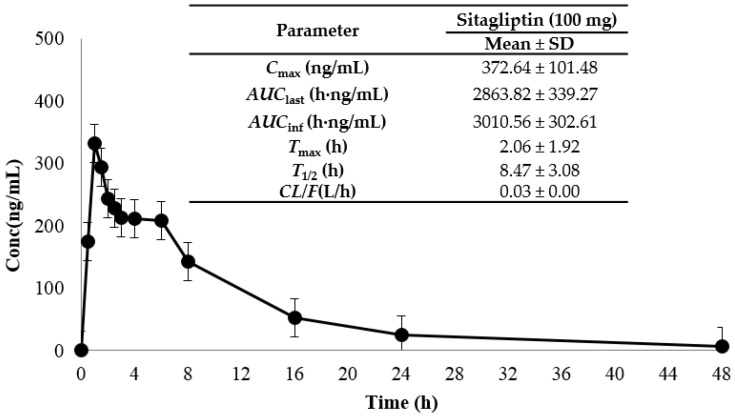
Mean (±SD) plasma concentration–time profile of sitagliptin (100 mg) following single oral administration in healthy male subjects (*n* = 8).

After administration, we recorded a mean *C*_max_ of 372.64 ng/mL, a *T*_max_ of 2.06 h, and an area under the concentration-time curve until the last measurable concentration (*AUC*_last_) of 2863.82 h·ng/mL (Figure 3). These compelling results affirm that our meticulously developed LC–MS/MS method is not only suitable but also excels in providing an accurate and reproducible pharmacokinetic evaluation of sitagliptin in human plasma.

### 2.4. Incurred Sample Reanalysis (ISR)

ISR was conducted to evaluate the consistency and reliability of the analytical method. According to regulatory guidelines [26,27], ISR is considered acceptable when at least 67% of reanalyzed samples exhibit a deviation within ±20% of their initial measurements. In this study, 62 samples were randomly selected from a total of 611 and reanalyzed using Stata/SE version 11.0. All selected samples met the established acceptance criteria for sitagliptin, confirming the reliability and reproducibility of the bioanalytical method.

## 3. Materials and Methods

### 3.1. Chemicals and Reagents

Sitagliptin (purity 99.4%) and its stable isotope-labeled internal standard, rac-sitagliptin-d4-HCl (purity 99.5%, isotopic purity 98.3%), were sourced from Sigma-Aldrich (Steinheim, Germany) and TLC Pharmachem (Markham, ON, Canada), respectively. All solvents used for analysis, including HPLC-grade methanol, acetonitrile, and methyl tert-butyl ether (MTBE), were obtained from JT Baker (Phillipsburg, NJ, USA). Reagents such as ammonium formate and formic acid were provided by Sigma-Aldrich. Ultrapure water was produced using the AQUAmax^®^ purification system from YoungLin Co. (Anyang-si, Korea). Blank human plasma was supplied by BioChemed Services (Winchester, VA, USA) and stored at −70 °C until use.

### 3.2. Liquid Chromatographic Conditions

Chromatographic analyses were expertly conducted using the Agilent 1200 system (Santa Clara, CA, USA), featuring an efficient autosampler, a precisely controlled thermostated column compartment, and a dual pump configuration. The detection of analytes was accomplished with the advanced API 4000 triple quadrupole mass spectrometer (AB Sciex, Framingham, MA, USA), which is equipped with an electrospray ionization (ESI) interface operating in positive ion mode. This sophisticated detection and quantification were carried out in multiple reaction monitoring (MRM) mode, ensuring fine analytical selectivity and sensitivity. System control and data acquisition were seamlessly managed through Analyst^®^ software version 1.6.2 (AB Sciex), allowing for a streamlined and reliable analytical process.

### 3.3. Mass Spectrometric Conditions

Mass spectrometric parameters were optimized to enhance the ionization and fragmentation efficiency of sitagliptin and its deuterated internal standard. Analyses were performed using an API 4000 triple quadrupole mass spectrometer. The ion spray voltage was set to 5500 V, and the source temperature was maintained at 550 °C. Nebulizer gases (GS1 and GS2) were adjusted to 55 psi, while the curtain gas (CUR) was set at 20 psi (Table 5). The collision gas pressure was maintained at 6 psi. Additional parameters, including declustering potential (DP), entrance potential (EP), and collision energy (CE), were individually optimized for each analyte. The selected MRM transitions used for quantification were determined during the method development stage.

### 3.4. Preparation of Calibration Standard Solutions and Quality Control Samples

Sitagliptin and IS reference standards were each dissolved in methanol to prepare stock solutions at 1 mg/mL concentrations. These stock solutions were diluted with 50% acetonitrile to prepare working standard solutions. The working solutions’ calibration curve (CC) concentrations were set at 50, 100, 500, 1000, 2500, 5000, and 10,000 ng/mL, while the quality control (QC) concentrations were 150, 3000, and 8000 ng/mL. The IS concentration was prepared at 1000 ng/mL. All working stock solutions were prepared and stored at −20 °C until use. Calibration standards and quality control samples were further diluted in human plasma to achieve final concentrations of 5, 10, 50, 100, 250, 500, and 1000 ng/mL for CC and 15, 300, and 800 ng/mL for QC. Consequently, the final calibration curve range was 5–1000 ng/mL.

### 3.5. Plasma Sample Preparation

Human plasma samples were removed from a −70 °C deep freezer and thawed at room temperature. After vortexing and centrifugation, 100 µL of plasma was transferred into a glass tube. To this, 20 µL of the IS (1000 ng/mL) was added, followed by the addition of 2 mL of MTBE as the extraction solvent. The mixture was vortexed vigorously for 10 min and centrifuged at 1699 g for 10 min at 4 °C. The upper organic layer (2 mL) was carefully transferred to a new tube and evaporated to dryness under a nitrogen stream at 40 °C. The dried residue was reconstituted with a total volume of 1 mL of a reconstitution solvent composed of 0.01% formic acid in DW and 100% ACN (1:1, *v*/*v*), vortexed for 10 min, and centrifuged again at 1699× *g*. A 5 µL aliquot of the clear supernatant was injected into the LC–MS/MS system for analysis.

### 3.6. Method Validation

Method validation was conducted according to the regulatory standards set by both the FDA and MFDS [26,27]. This process demonstrated acceptable performance across several parameters, including selectivity, LLOQ (5 ng/mL), calibration linearity, as well as the accuracy and precision of both intra-day and inter-day measurements. Additionally, factors such as recovery, matrix effects, and the stability of the analyte under various storage and handling conditions were also evaluated.

#### 3.6.1. Selectivity and LLOQ

Selectivity was rigorously evaluated through the analysis of six individual lots of blank human plasma, alongside pooled plasma samples, to uncover any potential interference from endogenous substances. Each sample was meticulously processed according to the detailed pretreatment protocol, and remarkably, no significant interference was detected at the retention times of sitagliptin or IS.

The LLOQ was established based on a signal-to-noise (S/N) ratio exceeding 10, with accuracy and precision at the LLOQ level falling within the ±20% acceptance criteria.

#### 3.6.2. Linearity and Carryover

Linearity was assessed using a weighted (1/x^2^) linear regression model across a calibration range of 5–1000 ng/mL. The calibration curve was defined by the equation y = ax + b, where y represents the mean peak area ratio of the analyte to the internal standard, x denotes the analyte concentration, a is the slope, and b is the y-intercept. A correlation coefficient (*r*^2^) of ≥0.99 was considered indicative of satisfactory linearity. Furthermore, all calibration standards were required to meet the acceptance criteria of accuracy within ±15% (85–115%), except for the LLOQ, which was accepted within a ±20% range (80–120%).

Carryover assessment was performed by injecting ULOQ (upper limit of quantification) and LLOQ samples along with double-blank samples. This test ensured that residual analyte from the highest concentration sample did not carry over and affect subsequent samples.

#### 3.6.3. Precision and Accuracy

The precision and accuracy of this method were rigorously evaluated by analyzing the concentrations of each sample through five replicates within the validation batch. For intra-day and inter-day testing, QC samples were prepared at four concentration levels: LLOQ (5 ng/mL), LQC (15 ng/mL), MQC (300 ng/mL), and HQC (800 ng/mL). These samples underwent analysis in five replicates over a span of three days, allowing for a thorough evaluation of accuracy and precision. According to our stringent evaluation criteria, the mean accuracy and precision should consistently fall within ±15% (85–115%) of the nominal value, with the exception of the LLOQ, which maintains a slightly broader range of ±20% (80–120%). This careful methodology underscores our commitment to ensuring the utmost reliability of our results.

#### 3.6.4. Recovery and Matrix Effect

Recovery and matrix effects were meticulously evaluated to understand the ion enhancement or suppression introduced by the matrix. To assess recovery, we focused on three distinct QC concentration levels: LQC (15 ng/mL), MQC (300 ng/mL), and HQC (800 ng/mL. This involved a thorough comparison of the mean analytical peak area from six replicates of extracted QC samples against the peak area of the same analyte concentration spiked into blank plasma after extraction. The evaluation of the matrix effect was equally critical. We contrasted the peak area of the QC concentrations in the standard solution with the peak area of the same analyte concentration spiked into the residue extracted from blank plasma. This comprehensive analysis highlights the intricate interplay between the analyte and its matrix, ensuring the integrity and reliability of our results.

#### 3.6.5. Stability

The stability of both stock and working solutions was evaluated at low and high QC levels (*n* = 3) under storage conditions of room temperature (25 °C) for 3 h and −20 °C for 7 h. All results were within the acceptable deviation limit of ±15% from the nominal concentrations. To assess the plasma stability of the analyte, low, medium, and high QC levels (*n* = 3) were tested under various conditions, including refrigerated storage at 4 °C for 24 h, deep-freezing at −70 °C for 7 h, three freeze–thaw cycles (between −70 °C and room temperature), and storage in the autosampler at 10 °C for 30 h. All measured concentrations under these conditions met the predefined acceptance criteria of ±15%, confirming the robustness and reliability of the analytical method.

### 3.7. Application to a Pharmacokinetic Study

This study procedures were conducted with the principles of the Helsinki Declaration (World Medical Association Declaration of Helsinki 2000) and Korean Good Clinical Practice guidelines. The study was approved by the Institutional Review Board (IRB) of Kyung Hee University Hospital (IRB No. KHUH 2023-07-039; approval date: 20 October 2023). The validated LC–MS/MS method was utilized in a clinical study to assess the pharmacokinetic properties of sitagliptin after the oral administration of a single 100 mg tablet. Eight healthy Korean male subjects participated in the study, each receiving a single oral dose of sitagliptin (Januvia^®^ 100 mg tablet, Jung-gu, Seoul, MSD Korea) while fasting. Blood samples were collected using EDTA-K_2_ anticoagulant tubes at various time points: pre-dose (0 h), and at 0.5, 1, 1.5, 2, 2.5, 3, 4, 6, 8, 16, 24, and 48 h following administration. Once collected, the blood samples were centrifuged at 3000 rpm for 10 min to separate the plasma, which was then transferred to labeled tubes and stored at −70 °C until analysis. All clinical procedures, including dosing, sampling, and analysis, were conducted consistently across all subjects.

### 3.8. Incurred Sample Reanalysis

ISR is performed to verify the reproducibility of the initial analytical results. In this study, approximately 10% of the total samples (62 in total) were randomly selected from both the absorption (peak) and elimination phases for ISR evaluation. The deviation between the original and reanalyzed concentrations was determined by comparing their mean values. According to regulatory guidelines, the ISR is considered acceptable when at least 67% of the reanalyzed samples fall within ±20% of the corresponding initial values.

### 3.9. Statistical Analysis of Bioequivalence

Pharmacokinetic parameters such as *C*_max_ (maximum plasma concentration), *T*_max_ (time to reach Cmax), *T*_1/2_ (elimination half-life), and *CL/F* (apparent oral clearance) were determined from individual plasma concentration–time profiles. Non-compartmental analysis for extravascular administration was performed using BA Cal 2007 software (MFDS, version 1.0.0). This analysis calculated parameters including *AUC*_last_ (area under the concentration–time curve from 0 to 48 h) and *AUC*_inf_ (area under the curve extrapolated to infinity). The statistical analysis of the pharmacokinetic data was conducted using K-BE Test II software (version 2.0.2), which was developed and distributed by the MFDS [26].

## 4. Conclusions

In conclusion, this study successfully developed a highly robust and efficient LC–MS/MS method for the accurate quantification of sitagliptin in human plasma. Utilizing only 100 μL of plasma and a simplified LLE procedure, the method achieved an excellent LLOQ of 5 ng/mL and a total run time of less than 2 min. Notably, the short analysis time allows for the rapid processing of multiple samples, making the method highly suitable for high-throughput analytical environments. Complying with internationally recognized validation guidelines, the method demonstrated outstanding selectivity, linearity, reproducibility, accuracy, extraction efficiency, matrix tolerance, and sample stability. The validated protocol was not only reliable in clinical pharmacokinetic assessments but also proved useful for various applications, including bioequivalence studies, therapeutic drug monitoring, and in-depth pharmacokinetic evaluations of sitagliptin. This innovative approach establishes a new standard in the field and is expected to contribute to future research advancements and improved patient outcomes.

## Figures and Tables

**Table 1:** Summary of regression parameters for the validated LC–MS/MS method for sitagliptin quantification in human plasma (*n* = 8).

Compound	Number	Linearity			
		Slope	Intercept	*r*	*r^2^*
Sitagliptin	1	0.00220	−0.000382	0.9983	0.9966
	2	0.00215	−0.000367	0.9998	0.9996
	3	0.00214	−0.000399	0.9997	0.9994
	4	0.00218	−0.001030	0.9995	0.9990
	5	0.00215	0.000336	0.9998	0.9996
	6	0.00208	0.000278	0.9973	0.9946
	7	0.00211	0.000968	0.9997	0.9994
	8	0.00209	0.001930	0.9993	0.9986

**Table 2:** Precision and accuracy of the LC-MS/MS method for determining sitagliptin concentration in human plasma, both intra- and inter-day.

Compound	Nominal Concentration (ng/mL)	Intra-Day (*n* = 5)					Inter-Day (*n* = 15)				
		Mean ± SD (ng/mL)			Precision (CV, %) ^a^	Accuracy (%) ^b^	Mean ± SD (ng/mL)			Precision (CV, %) ^a^	Accuracy (%) ^b^
Sitagliptin	5	5.06	±	0.19	3.72	101.18	4.91	±	0.49	9.87	98.17
	15	15.89	±	0.42	2.67	105.94	15.04	±	0.88	5.86	100.23
	300	289.35	±	4.41	1.52	96.45	293.57	±	6.67	2.27	97.86
	800	765.56	±	13.85	1.81	95.70	777.60	±	14.08	1.81	97.20

^a^ CV (%) = (standard deviation of the calculated concentrations/mean concentration) × 100. ^b^ Accuracy (%) = (predicted concentration/nominal concentration) × 100.

**Table 3:** Recovery of sitagliptin and IS from human plasma and the effects of the matrix (*n* = 6).

Compounds	Nominal Concentration (ng/mL)	Recovery (%)				Matrix Effect (%)			
		Mean ± SD			Precision (CV)	Mean ± SD			Precision (CV)
Sitagliptin	15	83.20	±	4.34	5.22	104.64	±	6.72	6.42
	300	81.99	±	2.19	2.67	105.43	±	2.65	2.52
	800	79.51	±	1.34	1.69	107.30	±	1.48	1.38
Sitagliptin-d4	1000	83.42	±	2.43	2.91	103.04	±	2.67	2.59

**Table 4:** Compellingly summarizes the stability of sitagliptin in both stock and working solutions, as well as in human plasma, across five distinct storage conditions at room temperature.

Stability Storage Condition	Sitagliptin Concentration								
	15 ng/mL (Mean ± SD, %)			300 ng/mL (Mean ± SD, %)			800 ng/mL (Mean ± SD, %)		
Solution stability (%)									
Stock Room temperature (3 h)	100.89	±	6.68				99.25	±	3.59
Working Room temperature (7 h)	100.19	±	7.76				95.93	±	1.51
Plasma Sample stability (%)									
Room temperature (7 h)	102.57	±	0.66	97.43	±	2.55	96.99	±	14.14
Refrigeration (7 h, 4 °C)	100.61	±	0.77	100.02	±	3.08	96.29	±	13.25
Freeze–thaw stability (3 Cycles)	103.73	±	1.08	98.57	±	2.12	97.71	±	7.24
Autosampler (30 h, 10 °C)	105.20	±	0.49	97.37	±	5.39	96.83	±	1.71
Deep freeze (7 h, −70 °C)	99.61	±	1.27	96.77	±	6.79	97.96	±	20.25

**Table 5:** Shows the mass conditions for the precursor ion of sitagliptin and IS.

Compounds	Ion Transition (*m*/*z*)	DP (V)	EP (V)	CE (V)	CXP (V)
Sitagliptin	408.2 → 193.0	91.0	9.0	30.0	40.0
Sitagliptin-d4	412.2 → 239.1	89.0	10.0	27.0	42.0

DP: declustering potential, EP: entrance potential, CE: collision energy, CXP: cell exit potential.

## Data Availability

The original contributions presented in this study are included in the article/Supplementary Materials. Further inquiries can be directed to the corresponding author(s).

## Supplementary Materials

The following supporting information can be downloaded at: https://www.mdpi.com/article/10.3390/molecules30142995/s1, Table S1: Summary of reported methods for sitagliptin.

## References

[1] García-Aguilar A., Guillén C. (2022). Targeting pancreatic beta cell death in type 2 diabetes by polyphenols. Front. Endocrinol..

[2] Gilbert M., Pratley R. (2020). GLP-1 analogs and DPP-4 inhibitors in type 2 diabetes therapy: Review of head-to-head clinical trials. Front. Endocrinol..

[3] Das D., Halder D., Bose A., Shaw T.K., Saha C., De P.K., Maji H.S., Pal T.K. (2022). Determination of Metformin and Sitagliptin in Healthy Human Volunteers’ Blood Plasma and Its Bioequivalence Study Under Fasting Condition. Int. J. Appl. Pharm..

[4] Drugs.com Sitagliptin Monograph. https://www.drugs.com/monograph/sitagliptin.html.

[5] Kalra S., Singh A.K., Das S., Pendurthi B., Dharmadhikari S., Ahire P., Khandhedia C., Markandeywar N., Mane A., Mehta S. (2025). Sitagliptin as an Add-on Therapy to Other Glucose-lowering Agents in Patients with Type 2 Diabetes Mellitus: A Narrative Review. J. Assoc. Physicians India.

[6] Saini K., Sharma S., Khan Y. (2023). DPP-4 inhibitors for treating T2DM-hype or hope? an analysis based on the current literature. Front. Mol. Biosci..

[7] DrugBank Sitagliptin (DB01261). https://go.drugbank.com/drugs/DB01261.

[8] Yin R., Xu Y., Wang X., Yang L., Zhao D. (2022). Role of dipeptidyl peptidase 4 inhibitors in antidiabetic treatment. Molecules.

[9] Texas Health and Human Service Sitagliptin (Januvia) Monograph. https://www.hhs.texas.gov/sites/default/files/documents/doing-business-with-hhs/provider-portal/facilities-regulation/psychiatric/monograph/sitagliptin-januvia-monograph.pdf.

[10] Philippine Food and Drug Administration Sitagliptin (JANUVIA) Product Information. https://verification.fda.gov.ph/files/DRP-11159_PI_01.pdf.

[11] Xie Y., Zhou Q., He Q., Wang X., Wang J. (2023). Opportunities and challenges of incretin-based hypoglycemic agents treating type 2 diabetes mellitus from the perspective of physiological disposition. Acta Pharm. Sin. B.

[12] Med Central, Sitagliptin—Oral. https://www.medcentral.com/drugs/monograph/187692-307001/sitagliptin-oral.

[13] Ashraf M., Shahzad M.N., Hayat M.M., Rahman J., Ejaz S., Altaf H., Nasim F.H. (2015). Development and validation of an HPLC method for the quantification of sitagliptin in plasma and tablet dosage form. Lat. Am. J. Pharm..

[14] Al Bratty M., Alhazmi H.A., Javed S.A., Lalitha K.G., Asmari M., Wölker J., El Deeb S. (2017). Development and validation of LC–MS/MS method for simultaneous determination of metformin and four gliptins in human plasma. Chromatographia.

[15] Khoja S.S., Patel L.J. (2021). Development and validation of new analytical LC-MS/MS method for the estimation of antidiabetic drugs ertugliflozin and sitagliptin in combined pharmaceutical dosage form. J. Pharm. Res. Int..

[16] Nirogi R., Kandikere V., Mudigonda K., Komarneni P., Aleti R., Boggavarapu R. (2008). Sensitive liquid chromatography tandem mass spectrometry method for the quantification of sitagliptin, a DPP-4 inhibitor, in human plasma using liquid–liquid extraction. Biomed. Chromatogr..

[17] Hess C., Musshoff F., Madea B. (2011). Simultaneous identification and validated quantification of 11 oral hypoglycaemic drugs in plasma by electrospray ionisation liquid chromatography–mass spectrometry. Anal. Bioanal. Chem..

[18] Pamu S., Patyar S., Thakkalapally L. (2021). Development and Validation of a Novel RP-HPLC Analytical Method for Sitagliptin Determination in Human Plasma. J. Pharm. Res. Int..

[19] Han X., Wang J., Huang J., Peng L. (2019). A Rapid and Sensitive Method for the Pharmacokinetic Study of Janumet (Sitagliptin and Metformin) Tablets by LC-MS/MS Coupled with Ion-Pair Solid Phase Extraction. Curr. Pharm. Anal..

[20] Loh G.O.K., Wong E.Y.L., Tan Y.T.F., Lee Y.L., Pang L.H., Chin M.C., Damenthi N., Peh K.K. (2020). Simple and rapid LC-MS/MS method for determination of sitagliptin in human plasma and application to bioequivalence study. J. Chromatogr. B.

[21] Lukka P.B., Tang W., Hammarstedt A., Conrad T., Heijer M., Karlsson C., Boulton D.W. (2024). Racial Comparison of the Pharmacokinetics and Safety of Fixed-dose Combination of Dapagliflozin/Sitagliptin in Western and Korean Healthy Adults. Clin. Ther..

[22] Moon S.J., Yu K.-S., Kim M.-G. (2020). An assessment of pharmacokinetic interaction between lobeglitazone and sitagliptin after multiple oral administrations in healthy men. Clin. Ther..

[23] Reddy S., Ahmed I., Ahmad I., Mukhopadhyay A., Thangam S. (2015). Development and validation of a method for simultaneous estimation of metformin and sitagliptin in human plasma by LC–MS-MS and its application in a bioequivalence study. J. Chromatogr. Sci..

[24] Scherf-Clavel O., Kinzig M., Stoffel M.S., Fuhr U., Sörgel F. (2019). A HILIC-MS/MS assay for the quantification of metformin and sitagliptin in human plasma and urine: A tool for studying drug transporter perturbation. J. Pharm. Biomed. Anal..

[25] Thao N.N.N., Hieu N.N., Loan T.T.T., Tuan N.D. (2020). Development, Validation, and Application for Simultaneous Assay of Metformin and Sitagliptin in Human Plasma by liquid Chromatography-Tandem Mass spectrometry. Syst. Rev. Pharm..

[26] Ministry of Food and Drug Safety (2013). Guideline on Bioanalytical Method Validation. https://www.mfds.go.kr/brd/m210/down.do?brd_id=data0010&seq=13054data_tp=A&file_seq=1.

[27] Food and Drug Administration (2018). Bioanalytical Method Validation Guidance for Industry. US Department of Health and Human Services. https://www.fda.gov/media/70858/download.

