# Supplementary material for: Development and Validation of a Highly Sensitive LC–MS/MS Method for the Precise Quantification of Sitagliptin in Human Plasma and Its Application to Pharmacokinetic Study
Source: Molecules. 2025 Jul 16;30(14):2995. doi: 10.3390/molecules30142995 (PMC12298822; doi:10.3390/molecules30142995)
Supplement: Supplementary file 1 [file molecules-30-02995-s001.zip › molecules-3710804-supplementary.pdf]

Supplementary Materials

Development and Validation of a Highly Sensitive LC–MS/MS Method for the Precise Quantification of Sitagliptin in Human Plasma and Its Application to Pharmacokinetic Study

Table S1. Summary of reported methods for sitagliptin

manuscript references	Year	Capacity (mg)	Calibration range (ng/mL)	LOD	Mobile phase	Sample preparation	Sample volume (µL)	Internal standard	Total run time (min)	Injection volume (µL)	Fragmentation (Q1 → Q3)	Flow rate (mL/min)	Analyte R.T. (min)
3	2022	×	4.68-1200	0.75	A: Milli-Q water containing 10 mmol Ammonium Acetate (pH =3.6), B: Acetonitrile containing 0.1% Formic Acid (pH =2.4)	Acetonitrile protein precipitation	600	Metoprolol	7	10	408.10 → 235.20	0.3	3.97
14	2017	×	10-500	9.33	0.01 M ammonium formate buffer (pH 3.0): acetonitrile (80:20 v/v)	Acetonitrile protein precipitation	100	Alogliptin	20	5	408.3 → 234.9	0.4	12.98
15	2021	100	100-3000	66.79	0.1% formic acid: acetonitrile (10:90)	×	×	×	6.5	5	408.1 → 234.95	0.6	3.2
16	2007	100	0.1-250	0.1	0.03% formic acid–acetonitrile (30:70, v/v)	LLE (MTBE:MC)	500	Fluoxetine	2	10	408 → 235	1.0, split (1:9)	2.8
17	2011	×	×	×	90:10 ammonium formate (0.005 M, adjusted to pH 3 with formic acid/ acetonitrile (eluent A) and acetonitrile (eluent B)	LLE (MTBE)	500	Vildagliptin-d3	10	5	408.2 → 235.0	0.3	1.45
18	2021	100	100-3200	28.64	acetonitrile: 0.5% triethanolamine (20:80) with pH 6.5	LLE (MC:diethyl ether)	1000	Rosiglitazone	10	8	×	1	5.232

19	2019	50	1-1000	1	80% (v/v) methanol in 5.0 mM ammonium formate water solution (pH 4.5)	Strata-× 33μ SPE extraction	100	Carbamaze-pine	4	5	408.3 → 235.1	0.2	2.8
20	2020	100	5-500.03	5	0.1% v/v formic acid and methanol (45:55, v/v)	Methanol protein precipitation	250	Diphenhydr-amine HCl	2	1	408.10 → 235.15	0.45	0.697
21	2024	100	2-1500	2	5 mM ammonium acetate and acetonitrile running in a 60:40 ratio over 3 min	Protein precipitation	×	Sitagliptin-d4	3	×	407.90 → 235.20	×	×
22	2020	100	1-1000	1	0.5% (v/v) formic acid in D.W (mobile phase A) and acetonitrile (mobile phase B)	Acetonitrile protein precipitation	100	Sitagliptin-d4	×	2	408.38 → 235.25	0.4	×
23	2014	100	3-800.5	0.75	20 mM ammonium acetate (pH 4.5±0.2): acetonitrile: 50: 50, v/v	Acetonitrile protein precipitation	100	Sitagliptin-d4	3.2	15	408.1 → 235.25	1.0, split (×)	1.37
24	2019	100	3.2-480	3.2	A: mixture of a 9 mM ammonium formate solution, formic acid and acetonitrile (450:0.25:50, v/v/v) B:80 mM ammonium formate solution, formic acid and acetonitrile (100:0.5:900, v/v/v) - Gradient	Acetonitrile protein precipitation	50	Sitagliptin-[^{2}H]$_4$	3.5	10	408.3 → 193.0	1.0, split (×)	1.26
25	2020	50	1-800	1	0.1% formic acid and methanol (45:55, V/V)	Methanol protein precipitation	1000	Phenformin	4	10	408 → 235	1.0, split (×)	1.29

- × indicates that it was not written in each reference.
